# Stable multilineage xenogeneic replacement of definitive hematopoiesis in adult zebrafish

**DOI:** 10.1038/srep19634

**Published:** 2016-01-18

**Authors:** Isabell Hess, Thomas Boehm

**Affiliations:** 1 Department of Developmental Immunology Max Planck Institute of Immunobiology and Epigenetics Stuebeweg 51 79108 Freiburg, Germany

## Abstract

Bony fishes are the most numerous and phenotypically diverse group of vertebrates inhabiting our planet, making them an ideal target for identifying general principles of tissue development and function. However, lack of suitable experimental platforms prevents the exploitation of this rich source of natural phenotypic variation. Here, we use a zebrafish strain lacking definitive hematopoiesis for interspecific analysis of hematopoietic cell development. Without conditioning prior to transplantation, hematopoietic progenitor cells from goldfish stably engraft in adult zebrafish homozygous for the c-myb^I181N^ mutation. However, in competitive repopulation experiments, zebrafish hematopoietic cells exhibit an advantage over their goldfish counterparts, possibly owing to subtle species-specific functional differences in hematopoietic microenvironments resulting from over 100 million years of independent evolution. Thus, our unique animal model provides an unprecedented opportunity to genetically and functionally disentangle universal and species-specific contributions of the microenvironment to hematopoietic progenitor cell maintenance and development.

The hematopoietic system has deep roots in animal evolution (for recent review, see^1^). This observation has stimulated the study of other vertebrate species apart from the traditional chicken and mouse models, most notably the zebrafish, to examine general and species-specific aspects of hematopoietic development and differentiation^2^,^3^,^4^,^5^. The zebrafish provides us with an ideal model system and is notable both for its ease of handling and the availability of a highly diversified genetic tool-box^6^. The zebrafish is a teleost and belongs to the most species-rich group of vertebrates inhabiting our planet; indeed, teleosts make up almost half of all vertebrates and are notable for their vast diversity in form and their ability to occupy the most extreme ecological niches^7^,^8^. Although it is conceivable that the analysis of diverse fish hematopoietic systems will shed light on the general principles and requirements of hematopoietic differentiation, with potentially great translational impact, no suitable experimental system has yet been developed to achieve this goal.

We recently described a zebrafish model for allogeneic hematopoietic cell transplantation^9^. It utilizes adult zebrafish homozygous for a missense mutation in the *c-myb* gene (c-myb^I181N^); this mutation causes failure of definitive hematopoiesis, thus obviating the requirement for pre-conditioning prior to transplantation of allogeneic hematopoietic cells^10^. Here, we have explored the possibility of using this model for xenogeneic transplantation as a first step towards examining the phenotypic diversity of fish hematopoietic systems. To this end, we chose goldfish as a species for our proof-of-principle studies. The goldfish is a member of the cyprinidae, the largest family of vertebrates, comprising more than 2,000 fresh-water species. Like carp, the goldfish *Carassius auratus* belongs to the subfamily of Cyprininae, whereas the zebrafish *Danio rerio* belongs to the subfamily of Danioninae. Recent phylogenetic analyses suggest that the last common ancestor of Cyprininae and Danioninae lived more than 100 million years ago^11^. This long period of independent evolution has led to widely diverging ecological preferences, the most notable of which is water temperature; whereas zebrafish adapted to tropical conditions with an optimal water temperature of 28.5 °C, goldfish tolerates water temperatures down to near freezing point. We therefore considered goldfish as an appropriate candidate to examine the feasibility of xenogeneic transplantation of hematopoietic cells.

There is considerable interest in examining the properties of vertebrate hematopoietic stem cells (HSCs). In particular, knowledge about the requirements for self-renewal and maintenance of HSCs could be harnessed for several therapeutic settings. The zebrafish has been successfully used for *in vivo* chemical screens aimed at identifying the molecular pathways regulating the number and maintenance of HSCs (for a recent example, see 12). However, while the zebrafish is clearly suitable for carrying out large-scale screens, it is not clear whether potential biological differences between the hematopoietic cells of fish and mammals may hinder the direct translational application of such results. Hence, it is important to explore the possibility of carrying out transplantations of xenogeneic HSCs into a zebrafish background with a view to generating an *in vivo* complement of existing *in vitro* models. As a first step in this direction, we explored the possibility of establishing a xenogeneic hematopoietic system in zebrafish. Our results indicate that the hematopoietic environment of the zebrafish supports the maintenance of xenogenic hematopoietic progenitor cells, opening up unprecedented opportunities for comparative studies on vertebrate hematopoiesis.

## Results

### Transplantation of goldfish whole kidney marrow into *c-myb* mutant zebrafish

**Figure 1:**
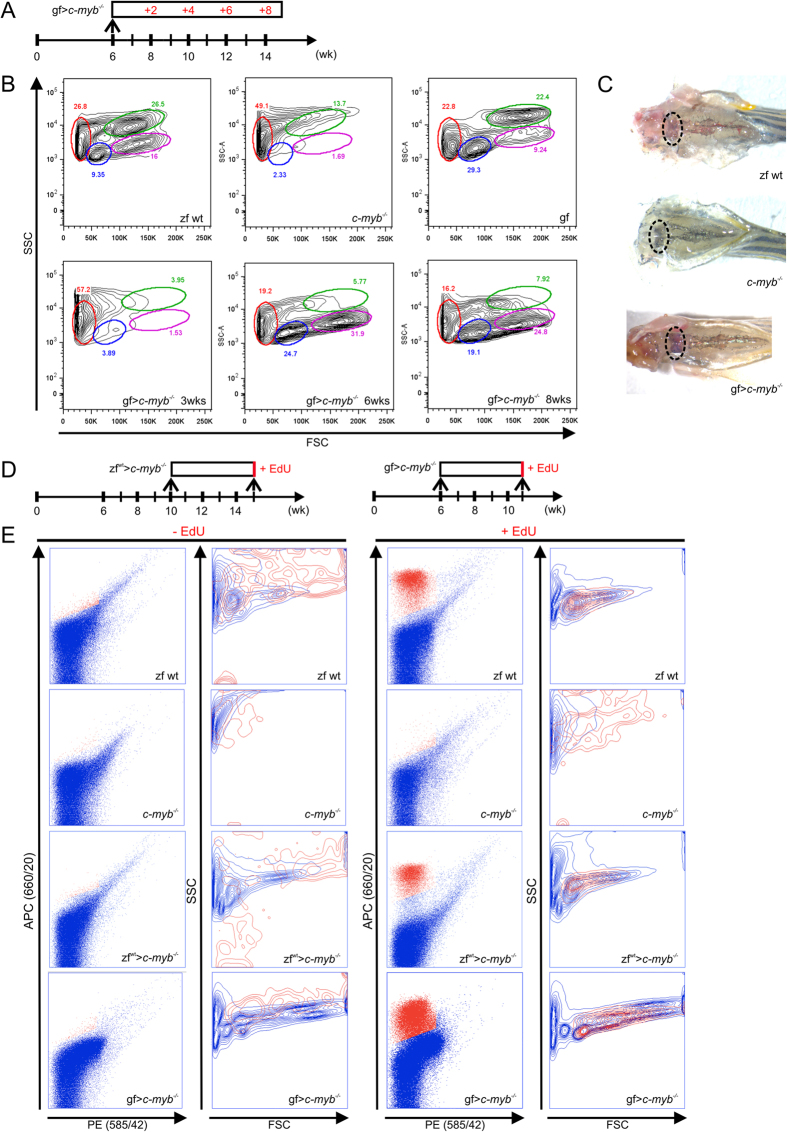
Reconstitution of hematopoiesis in adult c-myb^I181N^ zebrafish mutants after transplantation of goldfish kidney marrow cells. (**A**) Schematic outline. A total of 23 transplantations were carried out and analyzed at various time points; representative data are shown for each time point. (**B**) Flow cytometric analysis of whole kidney marrow cell populations of adult fish of various genotypes and species (top panels) and after transplantation of goldfish marrow cells into mutant zebrafish (lower panels); the time points indicate time periods after transplantation. Wild type zebrafish (zf wt), *c-myb* mutant (*c-myb*^−/−^), goldfish (gf) and *c-myb* mutant transplanted with goldfish whole kidney marrow cells (gf > *c-myb*^−/−^). The different cell populations in zebrafish kidney are identified by their forward (size) and side (granularity) light scatter characteristics: red, erythrocytes; blue, lymphocytes; green, myelomonocytic cells; magenta, progenitor cells. (**C**) Macroscopic representation of kidney of wild type zebrafish (left), zebrafish mutants (middle), and zebrafish mutants reconstituted with goldfish hematopoietic cells (4 weeks after transplantation; right), all 10 weeks old. The locations of the head kidneys are indicated by ovals. (**D**) Schematic outline of proliferation assay. (**E**) Flow cytometric analysis of whole kidney marrow cells isolated from fish of the indicated genotypes pulse-labelled with EdU, creating a red-fluorescent signal in cells. No signal is observed in control mock-pulsed cells (two leftmost columns). Proliferating cells are detectable only in wild type zebrafish kidney marrow and in mutant zebrafish reconstituted with either zebrafish or goldfish cells, but not in unmanipulated zebrafish mutants (third column from the left). The light scatter characteristics of EdU-positive cells (red) are superimposed on EdU-negative cells (blue) (second and fourth columns from the left). Representative data of corresponding biological replicates are shown (zebrafish wild type, n = 7; *c-myb*^−/−^, n = 5; zf > *c-myb*^−/−^, n = 3; gf > *c-myb*^−/−^, n = 3).

In a first set of experiments, we compared the extent of hematopoietic reconstitution after transplantation of zebrafish or goldfish cells over a period of several weeks (Fig. 1A). Following transplantation of approximately 4 × 10^5^ zebrafish kidney marrow cells per 6-week-old adult *c-myb* mutant recipient^10^, hematopoietic reconstitution was readily observable after two weeks, as described previously^10^ (see also Supplementary Fig. 4A). After transfer of approximately the same number of goldfish cells (3.3 ± 0.9 × 10^5^; n = 7), evidence of reconstitution became apparent at a later time point (Fig. 1B); nonetheless, in week 4 after transplantation, *c-myb* mutant recipients exhibited a reddish complexion (Fig. 1C), had lost their heart edema as a sign of reversal of anemia, and, as a consequence of improved tissue oxygenation, could swim much faster than their unmanipulated mutant siblings. When the cellular composition of the head kidney marrow was analyzed, the first distinct signs of repopulation were also evident at 4 weeks, and reconstitution was essentially complete at 6 weeks after transplantation (Fig. 1B). Of note, a shift in the relative distributions of different cell types - identifiable by their distinct forward and side light scatter characteristics - was evident; the most obvious change was the paucity of cells in the myelomonocytic gate (marked in green in Fig. 1B) relative to the original cell composition (Fig. 1B). Pulse-labelling with the nucleoside analogue EdU five weeks after transplantation (Fig. 1D) indicated that vigorous proliferation of hematopoietic cells occurred in the *c-myb* mutant recipients after transplantation of both zebrafish and goldfish cells, but not in unmanipulated *c-myb* mutant animals (Fig. 1E). Proliferation occurred primarily in progenitor and lymphocyte-like cells.

### No reactivation of endogenous hematopoiesis after transplantation

Next, we investigated whether the presence of wild type hematopoietic cells might stimulate *c-myb* mutant progenitor cells to undergo normal hematopoietic maturation, contributing to reconstitution after transplantation of goldfish hematopoietic cells. To exclude this possibility, we generated *c-myb* mutant fish additionally transgenic for *gata1:DsRed*^13^ and *ikaros:eGFP*^14^ reporters; the former transgene marks red blood cells, whereas the latter directs GFP expression in hematopoietic precursors and lymphoid cells. In *gata1:DsRed*; *ikaros:eGFP* double-transgenic *c-myb* wild type fish, numerous green fluorescent cells are clearly visible at 1 day after fertilization (dpf), whereas fewer fluorescent cells could be detected in *c-myb* mutants; this is compatible with the observation of failing definitive hematopoiesis in *c-myb* mutants^10^ (Supplementary Fig. 1A). At the same time point, red-fluorescent cells were detectable in both wild type and mutant fish, compatible with the presence of unimpaired primitive hematopoiesis^10^ (Supplementary Fig. 1A). In older fish, expression of *gata1:DsRed* and *ikaros:eGFP* reporters was confined to *c-myb* wild type fish, but absent in *c-myb* mutants; importantly, cellular fluorescence was not observed after transplantation of wild type zebrafish kidney marrow cells (Supplementary Fig. 1B and 1C). After the hematopoietic system had been fully reconstituted (Supplementary Fig. 1D), we used flow cytometry to examine the whole kidney marrow for the presence of red or green (i.e. mutant host) cells; however, none could be detected (Supplementary Fig. 1E–G). In adult *gata1:DsRed* and *ikaros:eGFP* wild type fish, *gata1* expression is particularly strong in the progenitor population, whereas *ikaros* expression predominates in cells with the light scatter characteristics of lymphocytes (Supplementary Fig. 1E). As expected, these cell populations are absent in adult *c-myb* mutants (Supplementary Fig. 1F). Similarly, no such cells could be found in *c-myb* mutants fully reconstituted with non-transgenic wild type kidney marrow cells (Supplementary Fig. 1G). These observations indicate that the *c-myb* mutation results in cell-intrinsically defective hematopoietic stem cell (HSC) maintenance and/or differentiation, preventing their contribution to hematopoiesis even after transplantation with wild type donor tissue. From an experimental point of view, this observation indicates that reconstituted hematopoietic and immune systems will consist entirely of descendants of transplanted HSCs.

### Multilineage hematopoietic reconstitution by goldfish cells

**Figure 2:**
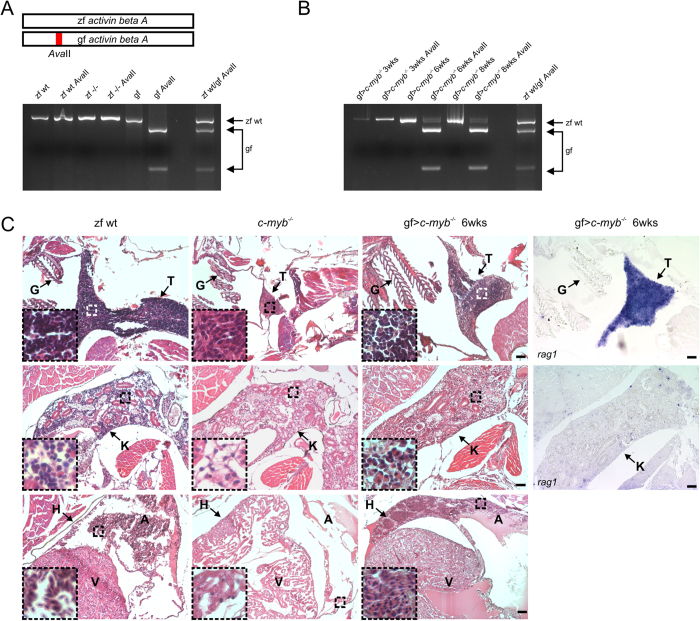
Multilineage hematopoietic reconstitution of *c-myb* mutant zebrafish after transplantation of goldfish whole kidney marrow cells. (**A**) Genotyping of whole kidney marrow cells of fish of indicated genotypes. The schematic at the top highlights the *Ava*II restriction site distinguishing zebrafish and goldfish sequences of the *activin beta A* gene (c.f. Supplementary Fig. 2A). Amplicons are shown before and after *Ava*II digestion of wild type zebrafish (zf wt), *c-myb* mutant zebrafish (zf^−/−^), and goldfish (gf). The *Ava*II digest of an amplicon generated from a 1+1 mixture of wild type zebrafish and goldfish cells is also shown on the right for reference. (**B**) Genotyping of whole kidney marrow cells of *c-myb* mutant zebrafish reconstituted with goldfish whole kidney marrow cells (gf > *c-myb*^−/−^) at several time points (in weeks) after transplantation. (**C**) Histological analysis of reconstituted *c-myb* mutant animals. Sections of the thymus (top panels), kidney (middle panels), and heart (bottom panels) are shown; insets represent 4× higher magnifications (haematoxylin/eosin staining) with the origin of selected regions indicated by broken squares (see also Supplementary Fig. 3). The fourth column indicates the results of RNA *in situ* hybridization using a *rag1* probe, detecting lymphoid progenitor cells actively rearranging antigen receptor loci. Wild type zebrafish (zf wt), *c-myb* mutant (*c-myb*^−/−^), and *c-myb* mutant transplanted with goldfish whole kidney marrow cells 6 weeks after transplantation (gf > *c-myb*^−/−^). All fish are 12 weeks old. Anatomical landmarks are indicated (G, gills; T, thymus; K, kidney; H, heart; A, atrium of the heart; V, ventricle of the heart). Scale bars, 50 μm.

In order to determine the relative contribution of goldfish cells in cell suspensions prepared from whole kidney marrow, we developed a simple PCR-based DNA typing assay; a small fragment of the highly conserved *activin beta A* genes of zebrafish and goldfish could be amplified using the same primer pair and readily distinguished by presence (goldfish) or absence (zebrafish) of an *Ava*II restriction site (Fig. 2A; Supplementary Fig. 2A). The results of this assay suggested the presence of goldfish cells in the repopulated zebrafish kidney marrow (Fig. 2B). We carried out detailed histological analyses to provide further evidence for the presence of ongoing hematopoiesis in *c-myb* mutants after transplantation of goldfish cells (Fig. 2C; Supplementary Fig. 3). Notably, our analyses demonstrated colonization of the thymus with strong *rag1* expression indicative of ongoing T cell development; similarly, B cell poiesis (as revealed by *rag1* expression in the kidney marrow) is readily detectable after transplantation (Fig. 2C). Finally, the pleomorphic appearance of peripheral blood cells (including red blood cells, lymphocytes and myeloid cells) was indicative of multilineage reconstitution (Fig. 2C; Supplementary Fig. 3). Cytological analysis indicated the presence of cells with the characteristically larger size of goldfish cells (Supplementary Fig. 2B); this size difference is also evident in the flow cytometric profiles (see Fig. 1B). Collectively, these results strongly suggest that transplantation of goldfish kidney marrow cells results in multilineage hematopoietic reconstitution of adult *c-myb* mutant zebrafish.

### Serial transplantation of xenogeneic hematopoietic progenitor cells

**Figure 3:**
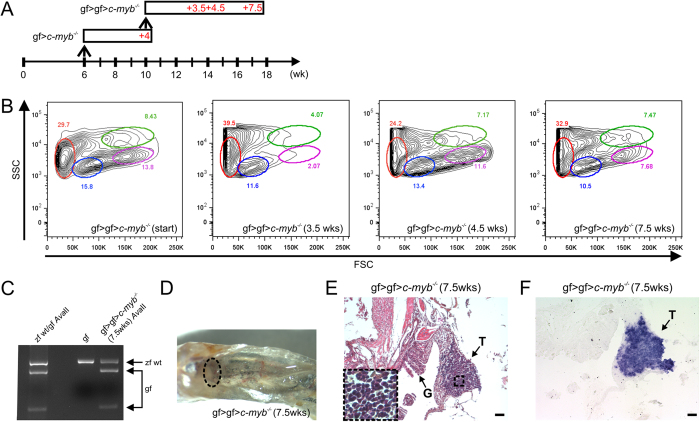
Serial transplantation of goldfish hematopoietic progenitors. (**A**) Schematic of successive transplantation experiment. A total of 8 transplantations were carried out and analyzed at various time points; representative data are shown for each time point. The first transplantation (gf > *c-myb*^−/−^) was carried out when c-myb mutants had reached the age of 6 weeks; the secondary transplantation (gf > gf > *c-myb*^−/−^) was carried out 4 weeks later. (**B**) Flow cytometric analysis of whole kidney marrow cells. The profile of the cellular source material for secondary transplant is indicated on the left (gf > gf > *c-myb*^−/−^ [start]); the other panels indicate the profiles for cells at various time points after the second transplantation. The different cell types are tentatively assigned (c.f. legend to Fig. 1). (**C**) Genotyping assay indicating the presence of goldfish cells in the whole kidney marrow derived from secondary transplantation. (**D**) Macroscopic view of kidney after secondary transplantation. The location of the head kidney is indicated by oval. (**E**) Colonization of the thymus (T) after secondary transplantation (haematoxylin/eosin staining); G, gills. (**F**) T cell development in the thymus (T) after secondary transplantation (RNA *in situ* hybridization with *rag1* probe). Scale bars, 50 μm.

Next, we assessed whether the hematopoietic system in *c-myb* mutants originating from goldfish cells would also support reconstitution after a second transplantation. To this end, we transferred all kidney marrow cells from one primary recipient to a secondary host (Fig. 3A). We established that recovery of hematopoiesis in secondary recipients took place with similar kinetics to those during primary reconstitutions; notably, the proportions of different cell lineages in the kidney are indistinguishable from those of the recipients of primary transplants (Fig. 3B–D). Moreover, the repopulated thymi displayed ongoing T cell development, as indicated by *rag1* expression (Fig. 3E,F). Collectively, multilineage reconstitution after secondary transplantation suggests that progenitor cells survive in the zebrafish kidney marrow environment.

### Interspecific competition of hematopoietic cells

**Figure 4:**
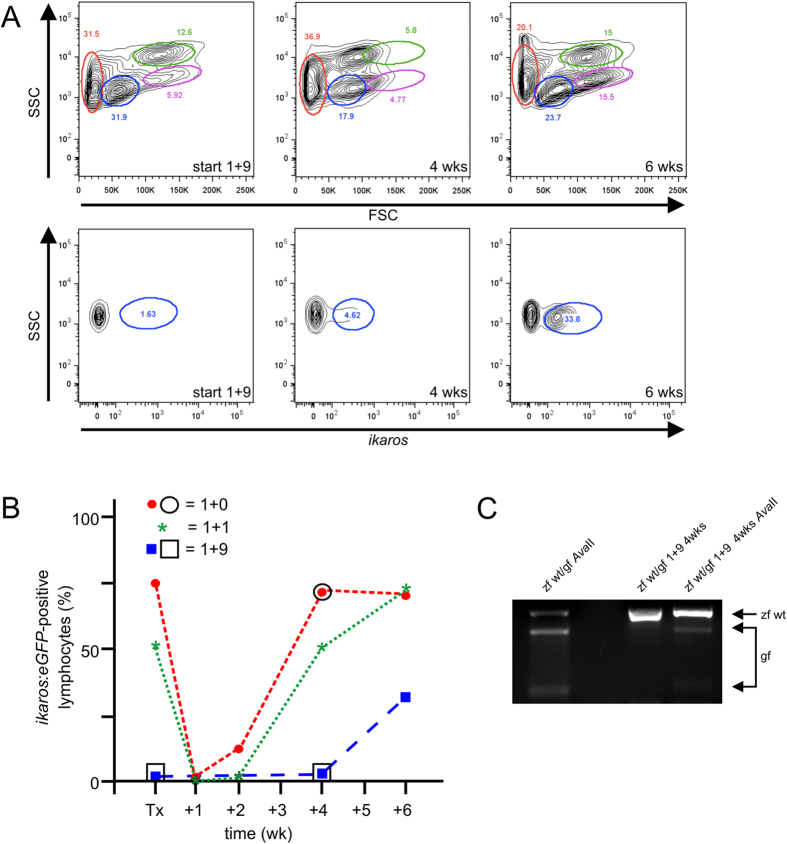
Competitive repopulation assay with excess goldfish cells. (**A**) Flow cytometric profiles after transplantation of a 1+9 mixture of zebrafish and goldfish cells, with different cell populations indicated (c.f. legend to Fig. 1). The starting material derived from *c-myb* wild type zebrafish transgenic for *ikaros:eGFP* reporter (1 part) and goldfish cells (9 parts) is shown in the top left panel; very few cells in the lymphocyte gate (blue) are GFP-positive (lower left panel). Analyses of whole kidney marrow cells at several time points after transplantation are also shown, indicating that complete reconstitution has not yet occurred after 6 weeks. Representative data for a total of five (1+9) transplantations are shown. (**B**) Time-resolved reconstitution of lymphocyte compartment, as measured by the percentage of GFP-positive cells over time after transplantation (in weeks). Summary of transplantation experiments for zebrafish whole kidney marrow cells only (1+0), and 1+1 or 1+9 mixtures of zebrafish and goldfish cells; each symbol represents the data of a single transplanted fish. (**C**) Genotyping assay indicating the presence of goldfish cells in whole kidney marrow 4 weeks after competitive transplantation of a 1+9 (zebrafish + goldfish) mixture.

The above experiments clearly indicate that under non-competitive conditions, goldfish hematopoiesis can be maintained in the zebrafish kidney marrow environment. However, the perturbed ratios of different cell types (*i.e*., lymphocyte *vs*. myelomonocytic cells) indicate possible species-specific differences in microenvironmental cues influencing the quantitative outcome of transplantation. If so, then such differences should become more pronounced in situations of competitive repopulation. To illustrate this potential use of the *c-myb* transplantation model, we carried out reconstitution experiments using transplants consisting of different ratios of zebrafish and goldfish cells. To monitor the kinetics of the reconstitution process, we employed zebrafish cells carrying the *ikaros:eGFP* transgenic marker, which is expressed in lymphoid and myelomonocytic cells. When we used a 1+1 mixture of zebrafish and goldfish whole kidney marrow cells, we established that hematopoietic reconstitution was eventually dominated by descendants of zebrafish HSCs, as indicated by the diagnostic proportion of GFP-positive lymphocytes (Supplementary Fig. 4). However, it is evident from a comparison of pure zebrafish transplants (Supplementary Fig. 4A) with the 1+1 mixtures (Supplementary Fig. 4B) that zebrafish hematopoiesis becomes established at a later time point and that goldfish myelomonocytic cells are at a disadvantage as compared to their zebrafish counterparts. Whereas zebrafish myelomonocytic cells had essentially outcompeted their goldfish counterparts 4 weeks after transplantation, lymphopoiesis was dominated by zebrafish cells only after 6 weeks (Supplementary Fig. 4C). When a 1+9 mixture of zebrafish and goldfish kidney marrow cells was used, reconstitution by zebrafish cells was even more delayed (Fig. 4A,B). However, goldfish cells proliferate alongside zebrafish cells in the initial phases of reconstitution, as indicated by the composition of the respective genomic DNA of kidney marrow cells (Fig. 4C). This observation argues against an immune-mediated depletion of either cell type and rather supports the notion that direct competition for niche space and/or growth and inductive factor activities contribute to the distorted outcome of chimeric reconstitution.

## Discussion

Maintenance of HSCs and their differentiation into the various blood cell lineages is regulated by a plethora of extrinsic cues such as growth factors and cytokines, some of which are known to be particularly species-specific in their action. In this context, it is surprising to find that hematopoietic reconstitution from transferred goldfish cells occurs in the zebrafish microenvironment. However, the relative paucity of myeloid cells in the xenotransplants relative to their frequency in the goldfish kidney marrow is one indication that the conditions for differentiation and/or survival of goldfish myeloid cells provided by the zebrafish microenvironment are not optimal. Nonetheless, our results demonstrate a surprising degree of functional conservation of hematopoietic supportive factors in species that have evolved independently for more than 100 Mya and that inhabit very different ecological niches. More specifically, our results predict that the homeostatic chemokines and cytokines supporting niche occupancy, colonization of primary hematopoietic (kidney) and lymphoid (kidney, thymus) organs and also differentiation of the major cell lineages are functionally compatible with the respective goldfish receptors. Our findings therefore provide important information concerning the molecular evolution of intercellular communication systems once the appropriate genomic and transcriptomic resources have been developed for the goldfish. Collectively, our observations suggest that the c-myb^I181N^ transplantation model described here could be developed for the study of hematopoietic systems of other fish species, facilitating comparative analyses of their cellular and functional characteristics. Our success bodes well for future attempts at transplanting into c-myb^I181N^ zebrafish hematopoietic cells from more distantly related organisms, such as jawless fishes and mammals, including humans.

So far, transplantation of non-tumorigenic xenogeneic cells into zebrafish was mostly performed using embryonic hosts to avoid the complications arising from immunological incompatibility (for instance, see^15^). Alternatively, hosts with compromised immune functions were used (for instance, see^16^,^17^). By contrast, the c-myb^I181N^ transplantation model does not require conditioning prior to transplantation. It is possible that the unmanipulated microenvironment contributes to the robust and stable engraftment of allogeneic^9^ and xenogeneic (this paper) hematopoietic cells. One interesting potential application of our model is the definition of the relative contribution of individual microenvironmental factors underlying the species-specific differences observed here. This could be done for instance by replacing zebrafish genes, individually or in combination, with orthologous sequences from other fish species or even more distantly related vertebrates; a second, but not mutually exclusive, alternative is the use of pharmacological or genetic screens to examine possible epistatic effects of the many different hematopoietic factors. Moreover, it should be possible, without having to consider interference by hematopoietic cells of the host, to examine the relative fitness of co-transplanted hematopoietic cell types, each differing in particular functionalities. Similarly to what was previously demonstrated for immunocompromised zebrafish hosts, our model could also be used for the transplantation of allogeneic and xenogeneic^16^,^18^,^19^ tumour cells to facilitate studies on their general biological properties and drug sensitivity profiles.

## Methods

### Animals

Zebrafish (*D. rerio*) wild type strains (TLEK and TL), the transgenic *ikaros:eGFP*^14^, the transgenic *gata1:DsRed*^13^ (a generous gift of Dr. T. Schwerte, University of Innsbruck, Austria) and the mutant *c-myb*^t25127^ 10 lines are bred at the Max Planck Institute of Immunobiology and Epigenetics. All procedures were conducted in accordance with institutional guidelines under a license (Az 35-9185.81/G-12/85) and approved by the local government (Regierungspräsidium Freiburg, Germany). In the past, homozygous *c-myb* mutants were housed in groups of 20 fish in separate tanks; recently, we have found that they can be maintained more easily by co-housing them with sentinel wild type fish, such as *slc45a2* albino fish^20^. In some experiments, transparent^21^ *c-myb* mutants were used (a generous gift of Dr. L. Zon, Harvard University).

### Transplantation procedure

Whole kidney marrow (WKM) cells from adult fish were harvested as described^9^ and finally resuspended at a concentration of 20,000 to 40,000 cells/μl in 0.9 × PBS supplemented with 5%FCS; cells were injected retro-orbitally in a volume of 10 μl per animal. After transplantation, zebrafish were kept at a water temperature of 22 °C (room temperature) instead of the usual 28.5 °C.

### Flow cytometric analysis

Flow cytometric analysis was carried out as described; the identities of cell populations were assigned according to their light scatter characteristics^22^. Profiles were recorded with an Aria II instrument, with the exception of the data shown in Supplementary Fig. 1; in this case an AriaFusion instrument was used.

### Fluorescence microscopy

Fluorescence microscopy was carried out as described^9^,^14^.

### Proliferation assay

To detect proliferating cells, 7 μg of 5-ethynyl-2′-deoxyuridine (EdU) in 10μl PBS solution (Click-iT^®^ EdU Flow Cytometry Assay Kits, Invitrogen Molecular Probes^®^) was injected retro-orbitally into wild type zebrafish, *c-myb* mutants and *c-myb* mutants carrying zebrafish and goldfish transplants, 4 weeks after primary transplantation. The analysis was performed 14–16 hours after EdU treatment. Staining was based on a click reaction using the Alexa Fluor 647^®^ dye and performed according to the manufacturer’s instructions.

### RNA *in situ* hybridization

This procedure was carried out as described^10^ using a zebrafish *rag1* probe^10^, which has a 87% nucleotide identity with goldfish *rag1* (c.f. Genbank accession numbers U71093.1 (zebrafish) und EF186007.3 (goldfish)).

### Genotyping

Genotyping for the *activin beta A* genes was carried out using primers 5′-GGATTATTTTGGACTGGCTCC and 5′-TTCAGATGCAGCATGTTGAGG; these bind to sequences that are identical for both zebrafish and goldfish orthologs and amplify fragments of almost identical size (zebrafish, nucleotides 248–598 in Genbank accession number BC129208; goldfish, nucleotides 134–479 in Genbank accession number AF169032) (Supplementary Fig. 2A). Amplicons were digested with *AvaII* (New England Biolabs) according to the manufacturer’s instructions and the products resolved on agarose gels. Genotyping for the presence of the *gata1:DsRed* transgene was carried out with primers 5′-CAAGGAGTTCATGCGCTTCA and 5′-TTCACGCCGATGAACTTCAC (amplicon size 360 bp); genotyping for the presence of the *ikaros:eGFP* transgene was carried out with primers 5′-TGGTGCCCATCCTGGTCGAG and 5′-GTCCTCGATGTTGTGGCGGA (amplicon size 488 bp).

## Additional Information

**How to cite this article**: Hess, I. and Boehm, T. Stable multilineage xenogeneic replacement of definitive hematopoiesis in adult zebrafish. *Sci. Rep.* **6**, 19634; doi: 10.1038/srep19634 (2016).

## Supplementary Material

Supplementary Information
