# Supplementary material for: Stable multilineage xenogeneic replacement of definitive hematopoiesis in adult zebrafish
Source: Sci Rep. 2016 Jan 18;6:19634. doi: 10.1038/srep19634 (PMC4726038; doi:10.1038/srep19634)
Supplement: Supplementary Information [file srep19634-s1.pdf]

Supplementary information for

Stable multilineage xenogeneic replacement of definitive hematopoiesis in adult zebrafish

Isabell Hess and Thomas Boehm

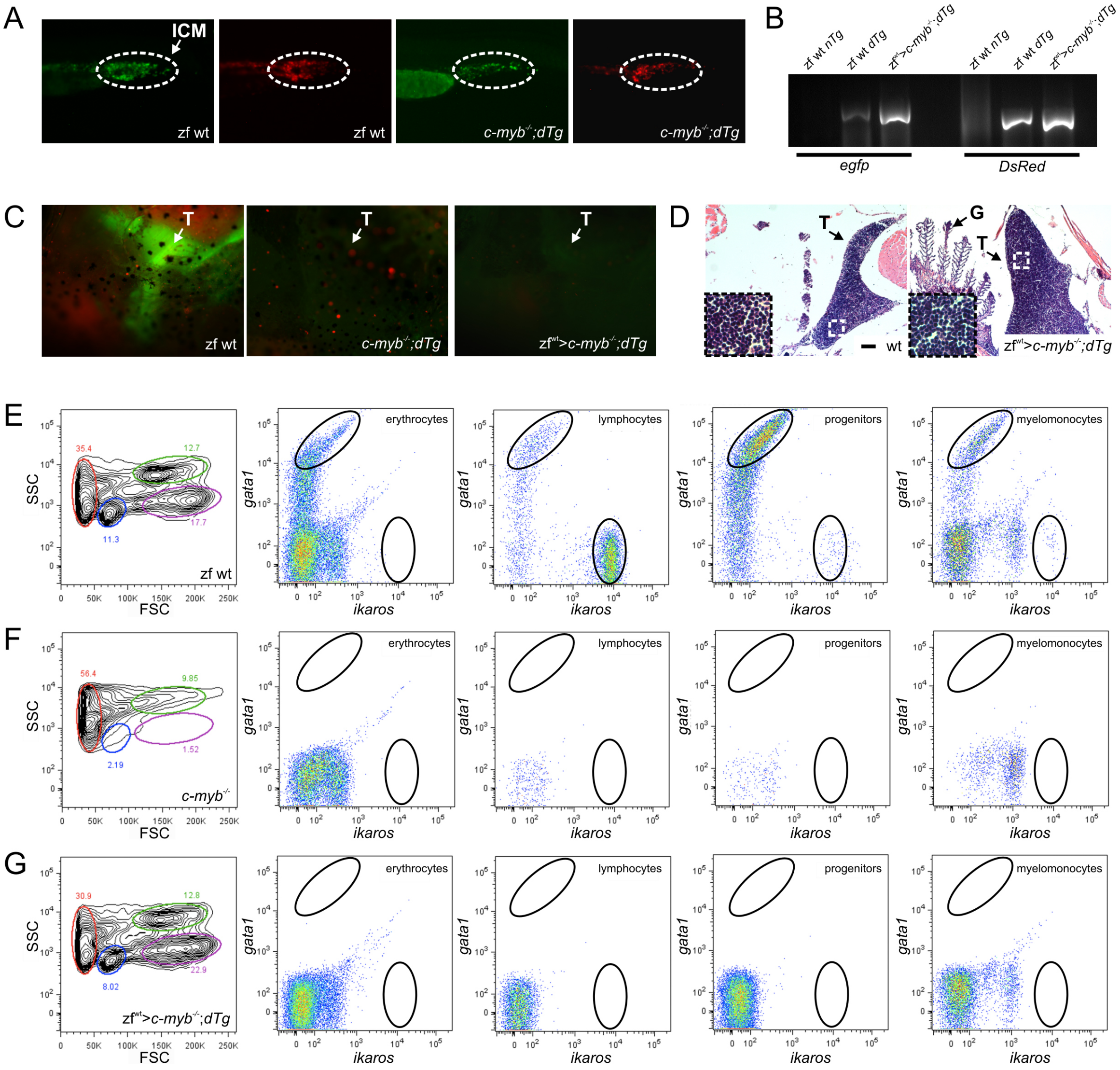

Supplementary Figure 1. *c-myb* mutant hematopoietic cells are intrinsically defective. (**A**) Fluorescence microscopic representation of wild type and *c-myb* mutant embryos transgenic for *gata1:DsRed* and *ikaros:eGFP* reporters at 1 dpf; the caudal hematopoietic tissue is shown. Note the reduced number of fluorescent cells in the intermediate cell mass (ICM; indicated by white oval) of mutant embryos. (**B**) PCR assay for the detection of transgenes in wild type (wt) or *c-myb* mutant (*c-myb*$^{-/-}$) fish; nTg, non-transgenic fish; dTg, double-transgenic for *gata1:DsRed* and *ikaros:eGFP* reporters. (**C**) Fluorescence microscopic appearance of the thymic region (T) of wild type (wt), *c-myb* mutant (*c-myb*$^{-/-}$) fish and *c-myb* mutant fish transplanted with wild type zebrafish whole kidney marrow cells 4 weeks after transplantation (zf^{wt}>*c-myb*$^{-/-}$), all fish

additionally double-transgenic for *gata1:DsRed* and *ikaros:eGFP* reporters (dTg). The bright red-fluorescent spots are caused by non-cellular background. (**D**) Reconstitution of hematopoiesis after transplantation of zebrafish wild type cells into *c-myb* mutants. Histological sections of thymi (T) of wild type and reconstituted animals are shown; G; gills. The inset is a 4x higher magnification indicating the dense repopulation with small lymphocytes. Abbreviations are as in (**A**) and (**B**). (**E-G**) Flow cytometric profiles of zebrafish of the indicated genotypes additionally transgenic for *gata1:DsRed* and *ikaros:eGFP* reporters, recorded using an AriaFusion instrument. Light scatter profiles are shown in the left-most columns; fluorescence profiles are indicated for the four major cell populations (red, erythrocytes, second columns; blue, lymphocytes, third columns; green, myelomonocytic cells, fourth columns; magenta, progenitor cells, fifth columns). (**E**) Flow cytometric profiles of wild type zebrafish (data representative of n=4). (**F**) Flow cytometric profiles of *c-myb* mutant zebrafish (data representative of n=3). (**G**) Flow cytometric profiles of *c-myb* mutant zebrafish reconstituted with non-transgenic wild type cells (data representative of n=6).

Scale bars, 50 μm.

A

```
                      F-primer
goldfish     GGATTATTTTGGACTGGCTCCCGCTTT--CCGCATCGATTTGACAAGCAAGCCAGCGCGG 58
zebrafish    GGATTATTTTGGACTGGCTCCCGCTTTTTCCGTATCGATCTGACAAACAAGCCAGTGCGC 60
             ***************************  *** ****** ******.******** ***
                                       AvaII
goldfish     CGACTGTGATCTTGGCAGCGGAGCAGAACAGGACCGAGGTGTGAGTCTGCCTTCACTCAG 118
zebrafish    CGACTGTGATCTTGGCAGAGGAGCAGAACAGGACTGAGGTGTGAGTCTGCCGTCACTCAG 120
             ******************.*************** **************** ********

goldfish     GATGTCCTCACTCACGCTCGTGAACAGGGGTACTGCTGCT---CTCCGGCTGTTTGTCAG 175
zebrafish    GATGTCCTCGCTCACGCTCGTG-ACAGGGGTGCTGCTGCTGCTCTCCGGCTGTTTGTCAG 179
             *********.************ ********.********   *****************

goldfish     GGGGCTGCTCACCCACTCCAGCCGAGAGTGGCTCTCAGGGGACGGGGAG-CCGGATGATC 234
zebrafish    GGGGCTGCTCGCCTACTCCATCTGATAGTGGCTCTCCAGGTGCGGGACGGCCCGATGATC 239
             **********.** ****** * ** **********..** .****..* ** *******

goldfish     CCGTGACCCCCTGCCCATCCTGCGCTCTAGCCCAAAGGCAGAAGGACTCAGAGGAGCAGA 294
zebrafish    CCGTGACCCCCTGCCCGTCCTGCGCTCTTGCCCAGAGGCCCAAGGACTCTGAGGAGCAGA 299
             ****************.***********:*****.****. ********:**********

goldfish     CCGACATGGTGGAGGCGGTCAAGAGGCACATCCTCAACATGCTGCATCTGAA 346
zebrafish    GTGACATGGTGGAGGCGGTCAAGCAGCACATCCTCAACATGCTGCATCTGAA 351
               *********************..***************************
                                                  R-primer
```

B

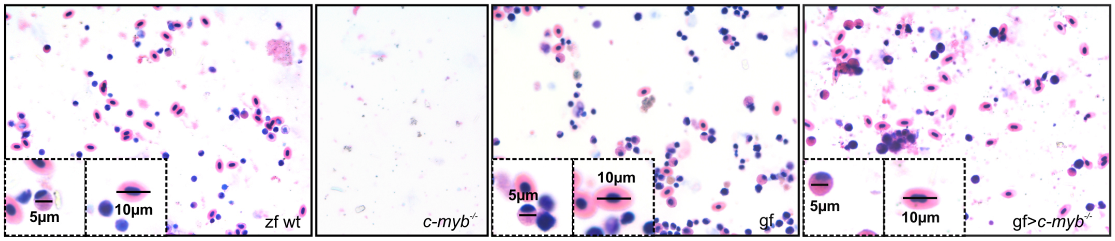

Supplementary Figure 2. Characterization of reconstituted *c-myb* mutants. (**A**) Sequence comparisons of zebrafish and goldfish *activin beta A* genes. The primer locations are indicated, as is the *Ava*II restriction site (GGWCC) in the goldfish sequence. (**B**) Giemsa staining of whole kidney marrow cells indicating the different sizes of zebrafish and goldfish cells. Zebrafish wild type (zf wt), *c-myb* mutant (*c-myb*$^{-/-}$), goldfish (gf) and *c-myb* mutant fish transplanted with goldfish whole kidney marrow cells 4 weeks after transplantation (gf>*c-myb*$^{-/-}$). The insets highlight individual cell types with scale bars indicated.

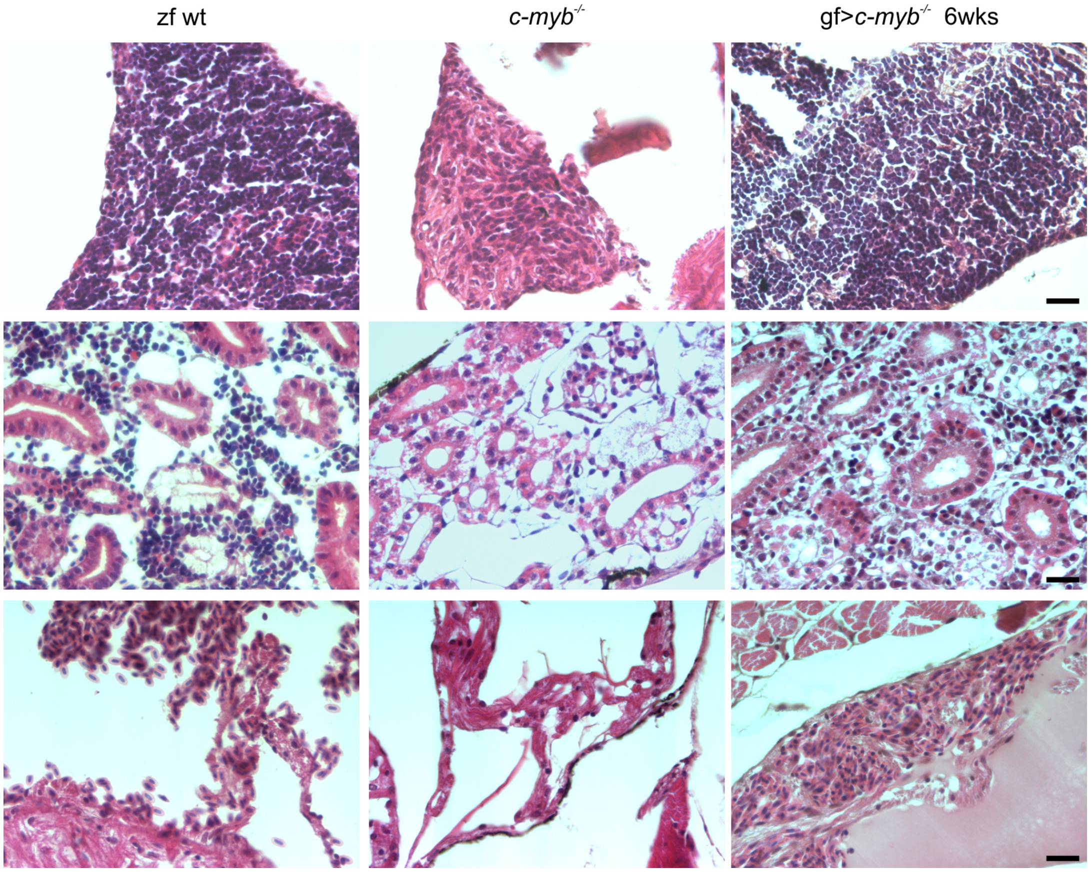

Supplementary Figure 3. Histological analysis of reconstituted *c-myb* mutant animals. Sections of the thymus (top panels), kidney (middle panels), and heart (bottom panels) are shown (see also Fig. 2) for wild type zebrafish (zf wt), *c-myb* mutant (*c-myb*$^{-/-}$), and *c-myb* mutant transplanted with goldfish whole kidney marrow cells 6 weeks after transplantation (gf>*c-myb*$^{-/-}$). All fish are 12 weeks old. Scale bar, 20 μm.

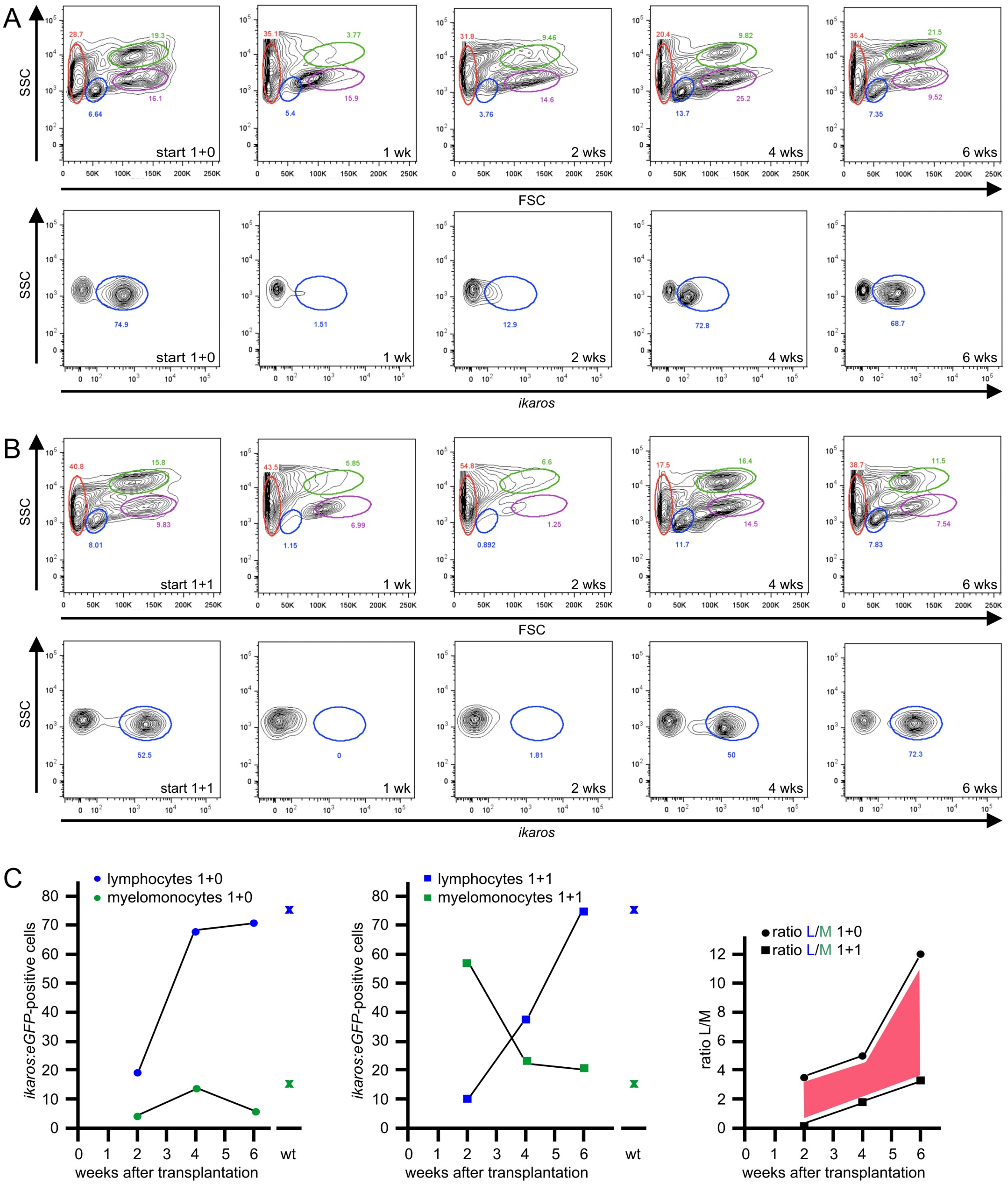

Supplementary Figure 4. Competitive repopulation assay. (**A**) Flow cytometric profiles of whole kidney marrow cells, with different cell populations indicated (c.f. legend to Fig. 1). The starting material derived from *c-myb* wild type zebrafish transgenic for *ikaros:eGFP* reporter without addition of goldfish cells (1+0 mixture) is shown in the top left panel; about three quarters of the cells in the lymphocyte gate (blue) are GFP-

positive (lower left panel). Analyses of whole kidney marrow cells at several time points after transplantation are also shown, indicating that reconstitution is essentially complete after at least 4 weeks. (**B**) Flow cytometric profiles after transplantation of a 1+1 mixture of zebrafish and goldfish cells as in (**A**); the light scatter gate for fluorescence analysis is set for zebrafish lymphocytes and thus does not capture all of the larger goldfish lymphocytes, explaining the somewhat greater percentage of GFP-positive cells in the starting material. Note that full reconstitution to approximately 75% GFP-positive cells in the zebrafish lymphocyte gate is not achieved until 6 weeks after transplantation. Representative data for a total of six (1+1) transplantations are shown. (**C**) Kinetics of hematopoietic repopulation in c-myb mutant fish transplanted with *ikaros:eGFP* transgenic zebrafish kidney marrow cells (1+0) and a mixture of *ikaros:eGFP* transgenic zebrafish and goldfish kidney marrow cells (1+1). The fractions of GFP-positive cells over time in the lymphocytic and myelomonocytic populations (identified by their light scatter characteristics) for the two donor cell conditions are shown in the left and middle panels, as are the ratios of fluorescent lymphocytic and myelomonocytic cells (right panel). The differences in the ratios (red area) give a measure of the relative advantages of zebrafish myelomonocytioc cells against their goldfish counterparts.
